# Transcriptomic Analysis of the Effect of Torin-2 on the Central Nervous System of *Drosophila melanogaster*

**DOI:** 10.3390/ijms24109095

**Published:** 2023-05-22

**Authors:** Yulia S. Vershinina, George S. Krasnov, David G. Garbuz, Mikhail V. Shaposhnikov, Maria S. Fedorova, Elena A. Pudova, Irina V. Katunina, Alexey B. Kornev, Nadezhda V. Zemskaya, Alexander A. Kudryavtsev, Elizaveta V. Bulavkina, Anna A. Matveeva, Natalia S. Ulyasheva, Zulfiya G. Guvatova, Artemiy A. Anurov, Alexey A. Moskalev, Anna V. Kudryavtseva

**Affiliations:** 1 Engelhardt Institute of Molecular Biology, Russian Academy of Sciences, 119991 Moscow, Russiaamoskalev@list.ru (A.A.M.); rhizamoeba@mail.ru (A.V.K.); 2 Center for Precision Genome Editing and Genetic Technologies for Biomedicine, Engelhardt Institute of Molecular Biology, Russian Academy of Sciences, 119991 Moscow, Russia; 3 Institute of Biology, Komi Science Center, Ural Branch of RAS, 167000 Syktyvkar, Russia

**Keywords:** RNA-Seq, TOR, neurodegeneration, neuroprotection, Torin-2

## Abstract

Torin-2, a synthetic compound, is a highly selective inhibitor of both TORC1 and TORC2 (target of rapamycin) complexes as an alternative to the well-known immunosuppressor, geroprotector, and potential anti-cancer natural compound rapamycin. Torin-2 is effective at hundreds of times lower concentrations and prevents some negative side effects of rapamycin. Moreover, it inhibits the rapamycin-resistant TORC2 complex. In this work, we evaluated transcriptomic changes in *D. melanogaster* heads induced with lifetime diets containing Torin-2 and suggested possible neuroprotective mechanisms of Torin-2. The analysis included *D. melanogaster* of three ages (2, 4, and 6 weeks old), separately for males and females. Torin-2, taken at the lowest concentration being tested (0.5 μM per 1 L of nutrient paste), had a slight positive effect on the lifespan of *D. melanogaster* males (+4% on the average) and no positive effect on females. At the same time, RNA-Seq analysis revealed interesting and previously undiscussed effects of Torin-2, which differed between sexes as well as in flies of different ages. Among the cellular pathways mostly altered by Torin-2 at the gene expression level, we identified immune response, protein folding (heat shock proteins), histone modification, actin cytoskeleton organization, phototransduction and sexual behavior. Additionally, we revealed that Torin-2 predominantly reduced the expression of *Srr* gene responsible for the conversion of L-serine to D-serine and thus regulating activity of NMDA receptor. Via western blot analysis, we showed than in old males Torin-2 tends to increase the ratio of the active phosphorylated form of ERK, the lowest node of the MAPK cascade, which may play a significant role in neuroprotection. Thus, the complex effect of Torin-2 may be due to the interplay of the immune system, hormonal background, and metabolism. Our work is of interest for further research in the field of NMDA-mediated neurodegeneration.

## 1. Introduction

The incidence of age-related diseases is increasing worldwide each year, placing a heavy burden on the health care system [1]. This has caused a growing need to search for the mechanisms responsible for these pathologies and for drugs that can prevent them.

The TOR signaling pathway, comprising two complexes (TORC1 and TORC2), binds extracellular signals and cellular nutrient status to regulate growth and metabolism during aging [2,3]. Deregulation of target TOR (target of rapamycin) is closely linked to a wide range of pathological conditions, including diabetes, cancer, neurodegenerative diseases, myopathies, inflammatory, infectious, and autoimmune conditions [4].

Normal TOR signaling has previously been shown to be essential for synaptic plasticity (NMDA-R-dependent) [4,5] and spatial learning [6]. TOR is also involved in the de novo synthesis of synaptic proteins [7,8] and is required for morphological changes associated with LTP induction in dendritic spines. Disruption of TOR signaling in the central nervous system is also thought to affect neuronal excitability and contribute to epileptogenesis [9].

On the other hand, there is incontrovertible evidence that reducing TORC1 activity—for example, through caloric restriction [10] or administration of rapamycin with food—increases longevity in model organisms, including *S. cerevisiae*, *C. elegans*, *D. melanogaster*, *M. musculus*, and other species [11,12]. Even mice treated with rapamycin from a later age lived longer [11,13]. Notably, primates also had increased longevity with fewer age-related abnormalities when caloric restriction began in adulthood. This includes preservation of brain grey matter volume [14]. Rapamycin and related compounds support protein synthesis in dendrites and their synapses [15,16], reduce Aβ and tau deposits in the hippocampus and improve learning and memory [17].

However, rapamycin has metabolic and immunological side effects, such as pneumonitis, stomatitis, poor wound healing, nephrotoxicity, and immunosuppression [18]. Rapalogs, such as temsirolimus and everolimus, as well as second-generation TOR inhibitors, such as Torin-2, significantly improve tolerability and are promising as therapies against aging and neurodegenerative diseases. Furthermore, the effective dose of Torin-2 is 400 times lower than that of rapamycin [19].

Most early studies looked at TOR inhibitors from two perspectives; numerous preclinical and few clinical studies examined the effects of TOR inhibitors on the body, including lifespan, immune function, and effects on the course of certain diseases [2,20,21]. The second point of consideration for TOR was to study its downstream substrates through a phosphoproteomic method, overwhelmingly on mammalian cell lines [22]. This method has limitations since in the body, many cell populations mutually influence each other; the immune systems of the body greatly affect the metabolism of introduced substances. In addition, when studying the effect of the substance on the nervous system, it is necessary to consider the presence of the BBB, which cannot be achieved when studying an isolated and immortalized cell line.

However, a transcriptome analysis of the brain under the influence of Torin-2 has not previously been performed on any model organism. At the same time, this analysis is of considerable interest due to the perspective of Torin-2 because of its possible geroprotective and neuroprotective properties.

Drosophila is a highly suitable genetic model to study the complex processes of aging and associated pathologies [23,24,25,26]. There are now many well-developed genetic methods that have made flies the leading model system for addressing questions about tissue-specific functional decline and interstitial interactions during aging [27,28,29,30].

For example, the roles of TOR [31] and age-associated memory pathologies [32] in *D. melanogaster* were previously studied in detail via the transcriptomic method, and the genes whose overexpression significantly affects lifespan (*dTsc1*, *dTsc2*, *dInR*, *dPTEN*, *dFOXO*, *daf-16*, etc.) were identified. In the present work, we evaluated the effect caused by a lifelong Torin-2-containing diet on the transcriptome of *D. melanogaster* heads in the context of aging.

## 2. Results

### 2.1. Selection of Concentrations and Longevity Analysis

**Figure 1:**
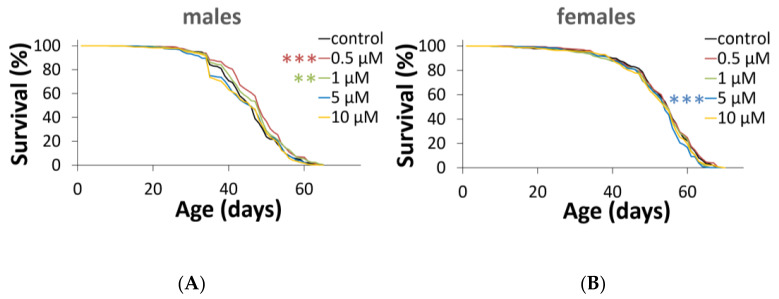
Effects of different concentration Torin-2 on lifespan in Drosophila males (**A**) and females (**B**). *** *p* < 0.001; ** *p* < 0.01; statistical significance of differences in survival curves is based on log-rank test; the number of flies (*n*) = 442 (control males); *n* = 490 (0.5 μM treated males); *n* = 484 (1 μM treated males); *n* = 460 (5 μM treated males); *n* = 507 (10 μM treated males); *n* = 470 (control females); *n* = 495 (0.5 μM treated females); *n* = 480 (1 μM treated females); *n* = 494 (5 μM treated females); *n* = 474 (10 μM treated females). Bonferroni correction was used for all multiple comparisons. All source data underlying the graphs and charts are presented in Supplementary Table S1.

In a series of experiments, Torin-2 at concentrations of 0.5 μM and 1 μM increased the median lifespan of males by 4% (*p* < 0.001, Fisher’s exact test). Increasing the concentration to 5 μM led to a decrease in the maximum lifespan of females of 3% (*p* < 0.05, Fisher’s exact test). In other variants of the experiment, there were no statistically significant changes in lifespan. The survival curves of males treated with Torin-2 at concentrations of 0.5 μM (*p* < 0.001, log-rank test) and 1 μM (*p* < 0.01, log-rank test) were shifted rightwards, while the survival curve of females treated with Torin-2 at concentrations of 5 μM was shifted leftwards (*p* < 0.001, log-rank test) (Figure 1 and Supplementary Table S1).

Thus, a concentration of 0.5 μM was chosen for the subsequent transcriptome analysis. However, we do not exclude that a further decrease in concentration may have had a more significant effect on a lifespan.

### 2.2. Age-Associated Transcriptome Changes in Insects Receiving Torin-2 and Controls

**Figure 2:**
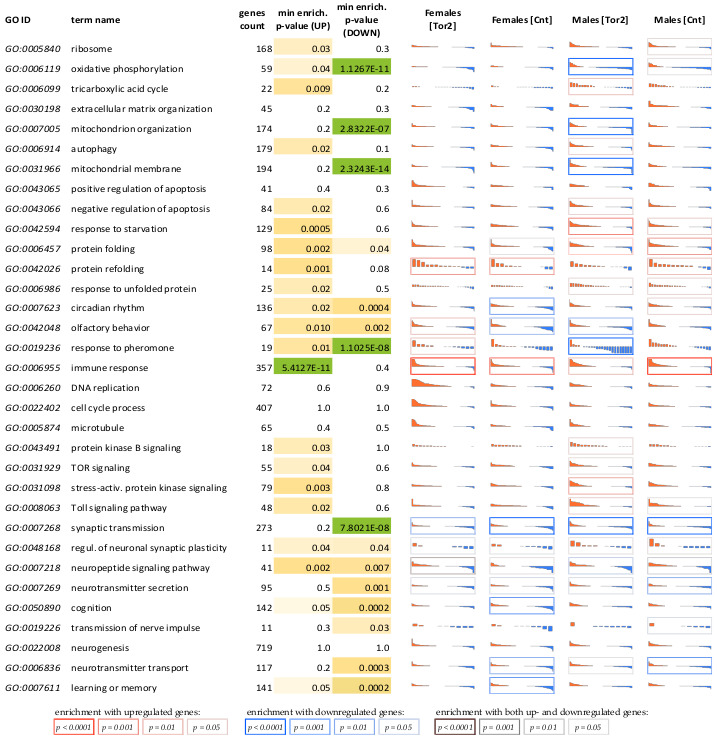
Age-associated (AA) changes in the expression levels of genes involved in various biological processes (Gene Ontology BP) that were mainly affected during aging in flies treated with Torin-2 or control flies. “GO ID”—Gene ontology term accession number. “genes count”—the total number of genes (in GO term), which have been included in the analysis. “min. enrich. *p*-value (UP)”—the most significant *p*-value for the overrepresentation test (for upregulated genes) among all comparisons. The same as for downregulated genes. Green-yellow-white color scale corresponds to these *p*-values (green—the most significant overrepresentation). Then, in the 4 columns on the right, in each cell there are collected Log2-values of age-associated expression level fold changes for all genes in a current GO term (Log2 values are sorted in descending order). Upregulated genes are in red, and downregulated genes are in blue. “Age-associated expression fold changes” are calculated from the slope of the line approximating the dependency of gene expression on age (by 3 points; in the ideal case, “Age-associated expression fold change” is 4 when gene expression in old flies is 4 times higher than in young flies and 2 times higher than in middle-aged flies). In each cell, the range on the vertical axis is from −2 (i.e., 4-fold decrease in expression) to +2 (i.e., 4-fold increase in expression). The color of the cell borders reflects the statistical significance (i.e., *p*-value) of the overrepresentation test results. Red borders indicate overrepresentation of upregulated genes (among members of the current biological process), blue borders indicate overrepresentation of downregulated genes, and black borders indicate overrepresentation of both types of genes.

First, based on RNA-Seq data, we identified the main transcriptomic patterns of natural aging in the control groups of flies (Figure 2). Natural aging of the male heads (most of which is allocated to the brain) was characterized by a dramatic induction of antimicrobial peptide genes (AMPs; *AttA*, *DtB*, *CecC*, *IM4*, *Prs*), genes responsible for sensory perception of light stimulus (*Nca*, *iraC*, *Rh3*, *tpr*), as well as decreased expression of genes involved in mitochondrial processes (*ACP*, *Cox5B*, *Cytc-p*, *NDB12*, *mtD4*), maintenance of cellular homeostasis (*comt*, *Cbp53E*, *Trxr-1*, *bgm*, *few*), hormone activity (*Proc*, *Crz*, *SIFa*, *Pdf*), neutrophil degranulation, and changes in extracellular matrix organization.

Natural female brain aging was associated with an increased expression of genes involved in the response to various biotic stimulus (*Lectin-galC1*, *TotX*, *PGRP-SB1*, *Sid*, *Stat92E*), oxidative stress (*chrb*, *Sid*, *Atg13*). Additionally, the expression of genes responsible for protein catabolism (*CG18467*, *Topors*, *CG11459*, *phyl*), members of pathways responsible for circadian rhythms (*Ilp3*, *GABA-B-R3*), lipid transport (*CG3009*, *ninaD*), steroid hormones (*DOR*), and peroxisome membrane transport (*Topors*) was significantly reduced in aging females. In general, the data obtained are consistent with previously published results on natural aging in Drosophila [33,34,35,36].

Among the most differentially expressed genes that showed increased expression under Torin-2 exposure in both sexes (LogFC > 2, i.e., four-fold or more changes in expression at FDR < 0.05), the following can be identified: *cort*, *Or42b*, *Or1a*, *CG11529*, E23, *bip1*, *Prat*, *Stip1*, *Bdp1*, *RfC4*, *SmD3*, *BCL7-like*, *arx*, *GstE1*, *Sema2a*, *mbf1*, and *Rassf*. Genes with decreased expression (LogFC < −2, FDR < 0.05) include *Sirt2*, *Mondo*, *hook*, *mRpS29*, *Atx2*, *Dbr*, *Golgin104*, *tn*, *HERC2*, *Srr*, *Eya*, *AMPKalpha*, *Ugt36E1*, *Lsm10*, *Ugt302K1*, *Brd8*, and *Sk2* (Supplementary Table S2).

It should be noted that in a direct comparison of Torin-2-treated flies with control insects (of the same sex and age), we did not find many of the observations that were seen when comparing age-related changes in gene expression profiles in the Torin-2 and control groups. One likely reason is that the magnitudes of many effects were small; the second reason is that the effect may be masked by 3′-bias.

In general, more differences in cellular pathways and biological processes affected by Torin-2 diet at the expression level were found for males rather than for females. Recall that the longevity analysis (Figure 1) showed a more significant effect of Torin-2 on males than on females. When directly comparing Torin-2-fed and control organisms, we identified 1372 differentially expressed genes (DEGs; *p* < 0.05) in males and only 344 DEGs in females. Of these genes, 405 passed the FDR threshold < 0.05 in males; only 29 did so in females at all three ages.

When directly comparing Torin-2-treated and control insects (males), we also found many genes with the increased expression that were involved in chaperone activity (including genes encoding for heat shock proteins Hsp83, Hsp27, Hsp26), L-amino acid catabolism, and cAMP synthesis. On the other hand, in males, we found the predominant upregulation of genes involved in the TCA cycle, bidirectional changes in the expression of other genes participating in oxidative phosphorylation, and glycolysis/gluconeogenesis. In females, the expression of these cell energy metabolism genes was slightly decreased. Additionally, in males, we noticed down-regulation of genes participating the response to pheromones.

Female flies under the influence of Torin-2 demonstrated upregulation of many genes participating in chromatin organization and remodeling, cell cycle, DNA replication, and immune response. However, these observations were not consistent between the replicates. For example, in the old female group, a predominant increase in cell cycle gene expression (GO:0007049) was observed only in 1 replicate out of 3. Similarly, in adult females, an increase in the expression of these genes was observed only in 1–2 replicates out of 3. In males, there was no predominance of increased or decreased expression in response to Torin-2 among the genes involved in the cell cycle and related processes.

Our results demonstrated that in males and females Torin-2 has a positive effect on the expression of genes participating the actin cytoskeleton formation. In addition, it is noteworthy that the expression of the gene encoding the serine racemase (*Srr*) is reduced under the influence of Torin-2 in all age and gender groups.

For two genes, we checked the expression changes using real-time qPCR: *Srr* and *JhI-21*. According to the RNA-seq data, *Srr* gene expression is decreased by 10%–40% in various groups, except for 4-weeks males, for which a 30% increase was observed. PCR data showed a similar result: decreased or unchanged expression in different sex and age groups, except for 4-weeks males, for which a 30% increase was also detected. Only partial consistency in the data was shown between RNA-seq and PCR for the *JhI-21* gene, whose expression was predominantly upregulated by Torin-2. The results are presented in Supplementary Figure S1.

### 2.3. Analysis of Gene Sets Specifically Associated with Age in Either the Torin-2 Group or the Control Group

**Figure 3:**
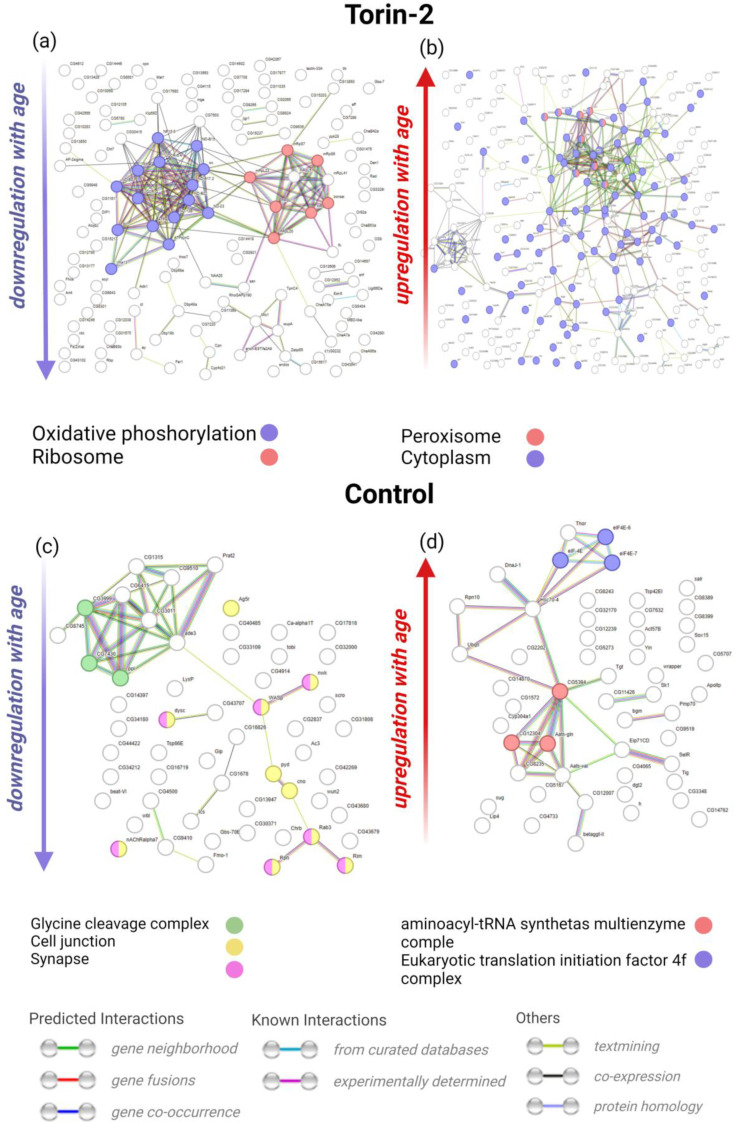
Inferred STRING networks (including physical interactions, co-expression, co-occurrence in databases, etc.) for genes whose expression was significantly associated with age in males: (**a**,**c**)—genes downregulated with age in Torin-2-fed and control flies, respectively (blue arrows down); (**b**,**d**)—genes upregulated with age in Torin-2-fed and control flies, respectively (red arrows up).

Based on intersection analysis, we selected 355 genes whose expression was associated with age only in *D. melanogaster* males receiving Torin-2 but not in any other groups. Similarly, 79 genes associated with age in control males were selected. Using the STRING database, we analyzed “association” networks for these subsets of gene separately for genes positively and negatively associated with age (Figure 3). These “associations” included physical interactions, co-expression, co-occurence in databases, etc.

Some of the protein products of genes whose expression was negatively associated with age in control flies but not in Torin-2 (i.e., Torin-2 at least partially prevented their age-dependent decrease in their expression) were localized in intercellular contacts, including synapses. Moreover, these genes were enriched with members of the glycine cleavage complex. At the same time, we observed age-dependent downregulation of many mitochondrial and ribosomal genes, specifically in theTorin-2 group but not the control group. This corresponds to the fact that Torin-2 tends to decrease the intensity of translation and cellular energy metabolism.

Supplementary Figure S2 shows the Venn diagram demonstrating the overlap between gene lists associated with aging across males and females, administered with Torin-2 and control groups. It should be said that the Venn diagram was created with fixed thresholds of statistical significance (for example, *p* < 0.05, |LogFC| > 0.3). Therefore, for example, the absence of a gene in the list of upregulated genes does not mean that its expression was not upregulated even to a small degree. Probably, it was indeed upregulated, but at a level lower than that to pass the threshold of statistical significance.

### 2.4. Expression of MAPK Pathway Genes (RNA-Seq Data)—Western Blot Analysis of Phosho- and Total ERK

We noted above that the expression of many genes involved in synaptic plasticity, learning, memory, and neurotransmitter synthesis decreases with age in *D. melanogaster* males and females (Figure 2). Along with this, Torin-2 can probably contribute to the maintenance of neuronal function in some age-related neurodegenerative diseases. To look at the neuroprotective effect of Torin-2 from a different point, we focused on the MAPK cascade. It has previously been shown that this pathway is involved in memory formation and cognitive functions.

According to the RNA-Seq data, the effect of Torin-2 on the expression of the MAPK pathway genes was quite different in different sex and age groups. Thus, we observed a predominant increase in the expression of MAPK member genes in young females (2-weeks old). The expression of 27 MAPK pathway member genes (GO:0000165; total of approx. 150 genes in term) was upregulated by at least 20% according to all three methods of RNA-Seq 3′-bias correction. The expression of six genes was upregulated by 40% or more: *sgg* (shaggy), *Spred* (Sprouty-related protein with EVH-1 domain), *rl* (rolled), *Alg-2* (Apoptosis-linked gene-2), *msn* (misshapen), *p38c* (p38c MAP kinase). Also noteworthy is the increased expression of *Crk*, *Mos*, *Ras85D* oncogenes, *Rala* (Ras-like protein A), *Pak* (p21-activated kinase), and *Gadd45*. A similar but less pronounced picture was observed for older females (6 weeks old) with other genes, and even less pronounced for older males.

In contrast, in middle-aged individuals (4 weeks old), there was a tendency towards a decrease in the expression of MAPK pathway genes. This was more pronounced in females. Thus, under the influence of Torin-2, the expression of the genes *p38a, p38b, p38c, rl, Gadd45*, *Cbl* proto-oncogene, *Mkp3,* and *Mkp4* (MAPK phosphatases) as well as others was reduced. Between females and males, the differences in the patterns of expression changes were quite significant. All these data are generally in good agreement between the three methods of RNA_Seq 3′-bias correction. The RNA-Seq data on the MAPK pathway genes are summarized in Supplementary Table S3.

The *rl* (rolled) gene encodes for the ancestral ortholog of mammalian ERK1/2. According to the RNA-Seq data, the increase of *rl* expression under the Torin-2 reached 2-fold in young females and 1.5-fold in old females, and it was decreased about 1.5-fold in young males.

**Figure 4:**
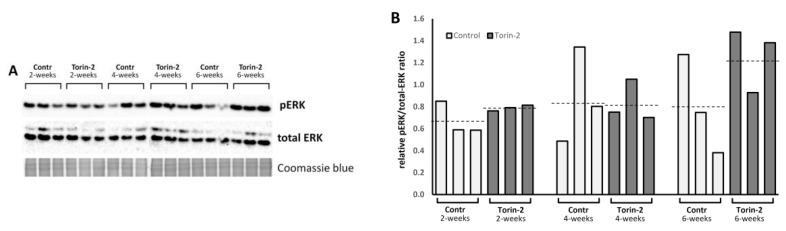
(**A**)—Immunoblot of proteins isolated from *D. melanogaster* male heads with anti-pERK and anti-total ERK antibodies. (**B**)—ratio of pERK to total ERK staining intensities. This ratio may be greater than 1 because of different affinities of anti-pERK and anti-total ERK antibodies. Dash lines indicate average value for each group.

Since in most cases the regulation of MAPK activity is carried out not only at the transcriptional but also at the post-translational level [37], we have performed a western blot analysis of the phosphorylation level of ERK, the most downstream node of the MAPK cascade in control and experimental groups of different age (Figure 4). Despite the large within-group variation in pERK/total-ERK ratios, there was a tendency for this ratio to increase with age in the groups treated with Torin-2: the largest increases in the pERK/total-ERK ratio were observed for old (6 weeks) flies. Perhaps the increased levels of phospho-ERK indicate a neuroprotective effect of Torin-2.

When comparing two pools of samples of all ages (control and exposed to Torin-2), we did not observe statistical significance here due to the small sample size (nonparametric Mann–Whitney test *p* = 0.17). The *t*-test was not used due to the presence of outliers.

## 3. Discussion

In the present work, we first evaluated changes in gene expression profiles in aging Drosophila, in control groups and insects with lifelong administration of Torin-2. We have identified changes in many cellular pathways and biological processes associated with age. Among them, we can list the pathways with a predominant increase in expression: humoral immune response (including genes encoding for antimicrobial peptides, AMPs), phototransduction, protein folding; as well as pathways with a predominant decrease in gene expression: oxidative phosphorylation, synaptic transmission; as well as various significant changes in metabolic pathways were observed. Many of the biological processes listed above are known to be associated with Drosophila aging [33,34,35,36,38].

The immune system is a complex network of many components that behave differently with age. It is known that Drosophila aging is followed with predominant decline of cellular immune response, decrease in the number of immune cells (hemocytes), and loss of their function [39]. On the other hand, data from many authors indicate that Drosophila overexpress AMPs genes with age, but data on their contribution to aging are ambiguous [40,41]. The function of APMs is not limited to direct pathogen elimination. They have a variety of other functions, such as control of gut microflora composition, regulation of nervous function and memory formation, participation in neurodegeneration, and also contributing to chronic age-associated inflammation [42].

According to our results, aging was also associated with at least slight overexpression of heat shock proteins (chaperones), mainly *Hsp26*, *Hsp27*, *Hsp68*, and *Hsp23*. In turn, in males and females, Torin-2 induced even greater expression of heat shock proteins *Hsp83*, *Hsp27*, and *Hsp26* according to RNA-Seq data. The family of heat shock proteins is heterogenous in its functions. For example, small Hsp (Hsp26, Hsp27) contribute to the folding of larger chaperones [43]. In turn, Hsp83 (Hsp90 homologue in mammals) binds to substrate proteins at a late stage of folding [44]. The Hsp family of proteins has previously been found to be important in memory formation and prevention of age-associated neurodegenerative pathogenesis [45,46,47,48,49]. Chaperones enhance the refolding of misfolded proteins and thus counteract their aggregation [50].

Some members of the Hsp family are known to be overexpressed with age [38,51], helping to control aging-associated proteotoxicity, accumulation of misfolded/denatured proteins exhibiting beneficial effects on the aging process [52]. Thus, as it was shown for genetically evolved strains of flies, higher levels of *Hsp22*, *Hsp26*, and *Hsp27* expression are strongly associated with increased longevity [53,54], but the data on *Hsp70* are ambiguous [55].

It has also been shown in previous studies that Hsp83 shown to physically bind to the insulin receptor InR and activate the InR/PI3K/Akt pathways in the nervous system. Hsp90 is considered a previously unidentified regulator of the critical neural stress sensor (DLK) which controls axonal regeneration, degeneration, and neurological disease [56]. It should be noted here that it is through Akt that the negative feedback loop of rapamycin administration is activated [57], which neutralizes the effect of rapamycin with prolonged exposure. Thus, exposure to Torin-2 probably activates the Akt pathway from two directions—directly as a downstream substrate of TORC2 and by activation through Hsp83.

The increase in aberrantly folded proteins in the brain of senescent Drosophila shown in earlier works [58] leading to increased neurotoxicity [50,59] may be compensated in our case by increased small Hsp levels and probably by autophagic processes, which unfortunately were not detected in our experiment as significantly increased by exposure to Torin-2.

When analyzing the obtained DEGs using the STRING (https://string-db.org/, accessed on 24 April 2023), it was shown that protein products of many genes with increased expression under the influence of Torin-2 are localized in the intercellular space and peroxisomes (Figure 3). This may also indicate the natural activation of gluconeogenesis in the TOR-inhibited cell.

Previously, in a study of the transcriptome profile of Drosophila, it was shown that down-regulation of mitochondrial genes is a feature of aging that is inherent to hydrogen peroxide and ionizing radiation [38].

The inhibition of TOR, the central regulator of growth and metabolism, activates stress stimuli pathways (for example, increased expression of genes participating starvation response pathway in Torin-2-treated flies). This may lead to an increase in Ca^2+^ and cAMP concentrations in the cytosol of nerve cells and activation of the Toll immune response pathway. The expression of many genes participating Toll pathway is elevated during aging, especially in males treated with Torin-2. Thus, the increased expression of antimicrobial peptides (AMPs) in the case of Torin-2 treatment may have a different origin than in natural aging [60].

A significant difference between males and females was revealed in the expression profiles of genes participating reproductive behavior and response to pheromones. These genes were predominantly downregulated in males and upregulated in females in response to Torin-2, and vice versa. Indeed, numerous studies have previously demonstrated differences in the pathogenesis of neurodegenerative diseases depending on sex. Estrogens have been shown to have anti-inflammatory activity [61], and microglia immune reactivity is differentiated sexually at very early stages of development [62]. Specifically, androgens have been shown to be responsible for immunosuppression in males after injury and immunostimulant in females [63,64,65,66].

In males, courtship is driven by vision, whereas females receive a combined sensory signal from odor and moisture [67]. In our results, however, the strongest changes in the response to Torin-2 in males were manifested by a decrease in genes responsible for male behavioral activity and pheromones.

Sex hormone receptors can induce a variety of signaling cascades ranging from genomic transcription to intracellular signaling pathways that are dependent on cell state [68]. Thus, the differences in the effect of Torin-2 on males and females may be due to the cross-influence of hormonal background, metabolism, and immune function [69].

Because of the difficulties we encountered in processing the results (i.e., 3′-bias), we cannot speak with certainty about the expression levels of mitochondrial genes under the influence of Torin-2 in females. The downregulation of these genes in males detected in our experiment may be the basis for additional investigation.

That said, there remains no doubt that Torin-2 altered the proinflammatory profile of the fly nervous system and that it did so more strongly in males.

In the nervous system, the main effector cells for innate immunity (*D. melanogaster* only has an innate immune response), inflammatory cytokines, and AFC [70,71,72] are glial cells (microglia). It is the activation of microglia that is responsible for the neurotoxic effect during aging.

**Figure 5:**
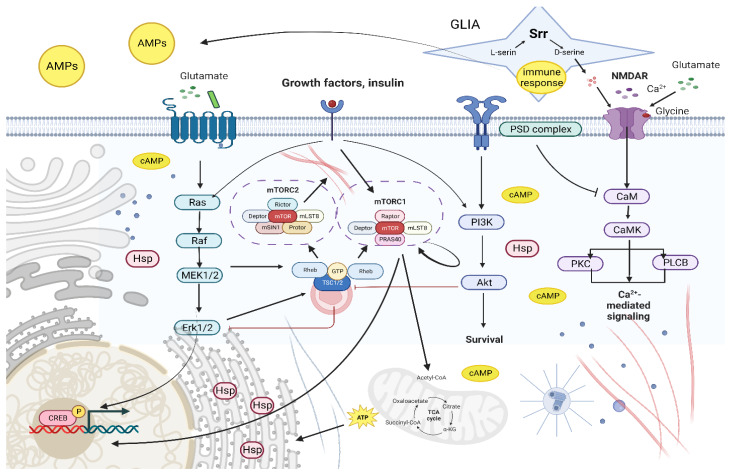
Generalized scheme of interaction between the participants of the processes possibly involved in the response of the *D. melanogaster* to the effect of Torin-2 (The figure was prepared using software provided by Biorender.com, accessed on 25 January 2023).

We hypothesize that Torin-2 may have some effect on the glycine site of the NMDA (N-methyl-D-aspartate) receptor (NMDAR), contributing to the normalization of calcium levels in the postsynaptic space. In microglia cells, the NMDAR responds to various cytokines. D-serine is an endogenous co-agonist in the glycine site of NMDA receptors and is required for receptor/channel opening [73,74]. In the nervous system, L- to D-serine is converted by the enzyme serine racemase (Figure 5). This protein is encoded by the *Srr* gene. We also found a consistent downregulation of the *Srr* gene under the chronic administration of Torin-2 in various sex-age groups.

D-serine production and release are strongly regulated, and its concentration is maintained within a narrow range. Chronic elevated D-serine levels have been shown to be associated with NMDAR-mediated neurotoxicity [75,76,77], whereas abnormally reduced levels are associated with impaired functional plasticity and memory deficits [78]. Determining the balance of L-serine and D-serine in pathologies of the nervous system and in exposure to metabolic altering substances is also of considerable current interest [77,79,80].

The link between all the processes described is schematically illustrated in Figure 5.

Typically, increased levels of Hsp and cAMP are associated with activation of the mitogen-activated protein kinase (MAPK, an upstream regulator of p38) pathway, which is important in the pathogenesis of neurodegenerative diseases.

According to the RNA-seq data, the expression of various MAPK pathway genes (GO:0000165) under treatment with Torin-2 exhibited different behavior in different sex–age groups. Thus, young and old females, as well as old males, showed a predominant increase in the expression of some genes of the MAPK pathway. In middle-aged females (and—to a lesser extent—males), there was a predominant decrease.

Thus, we were interested in the MAPK pathway and decided to check how Torin-2 presumably affects its activity level. In particular, its activity is linked with the content of the active phosphorylated form of ERK (pERK). Western blot analysis showed that the pERK/total-ERK ratio tends to increase under Torin-2 diet only in 6-week-old males (Figure 4) but no other age groups. Only males were included in the analysis, because only males were shown to have the effect of Torin-2 on longevity. This does not contradict RNA-seq data showing that expression of MAPK pathway members was, on average, elevated in older males.

In this case, we can only speak of a trend since there is large intra-group variation. Nevertheless, one may hypothesize a possible neuroprotective effect of Torin-2 that is realized through activation of MAPK cascade.

Summarizing all of the above, Torin-2 may be able to modulate the activity of many signaling proteins that participate in various regulatory cascades since it affects one of the central cellular regulators, TOR. On the basis of our findings, we can make the assumption that Torin-2 may exert a noticeable effect on the central nervous system in maintaining cognitive abilities in *D. melanogaster*. However, this question requires further research. The complex effect of Torin-2 may be due to the interplay of the immune system, hormonal background, and metabolism, which is the main target of the TOR pathway.

## 4. Materials and Methods

### 4.1. Drosophila Strain and Experimental Conditions

*D. melanogaster* wild-type Canton-S line (#64349, Bloomington, IN, USA) was used in the experiments. Newly eclosed flies were collected and reared in food medium containing 1000 mL water, 7 g agar, 8 g yeast, 30 g sugar, 30 g semolina, and 3 mL propionic acid. Males and females were kept separately in a Binder KBF720-ICH climate chamber (Binder, Tuttlingen, Germany) at 25 °C and 60% humidity, with light/dark alternation (12 h/12 h). Flies were kept in narrow Drosophila vials (Genesee Scientific, San-Diego, CA, USA) at a density of 30 individuals per vial and 5 vials per experimental and control group. Flies were transferred to the fresh medium twice a week.

**Figure 6:**
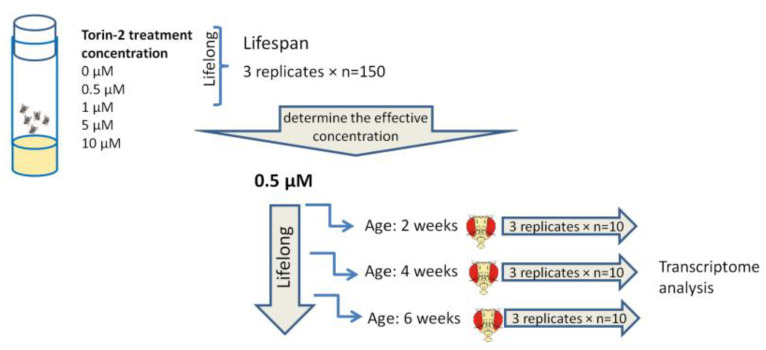
The experimental design of the study. A series of pilot experiments was performed to determine the concentration of Torin-2 that would have the best effect on longevity. Next, the selected optimal concentration of Torin-2 (0.5 μM) was used for lifelong feeding of flies for subsequent transcriptome analysis.

The overall experimental design is presented in Figure 6. A series of pilot experiments were performed to determine the concentration of Torin-2 that would have the best effect on longevity. Flies were kept on the nutrient medium spread with the paste of hydrolyzed yeast containing Torin-2 at concentrations of 0.5, 1, 5, and 10 μM (per 1 L) during their lifetimes (from imago hatching to death). Control flies were kept without Torin-2. Dead flies were collected daily. The experiment to evaluate the effect of Torin-2 on longevity was performed in 3 replicates. The selected optimal concentration of Torin-2 was used in the next experiment, lifelong feeding of flies for transcriptome analysis.

### 4.2. Transcriptome Sequencing

For transcriptomic analysis, the flies were fed with Torin-2 at a concentration of 0.5 μM. Flies at the ages of 2 (young), 4 (adult), and 6 weeks (old) were collected, and their heads were manually separated from the bodies and immediately frozen in liquid nitrogen. Three replications of the experiment for each group were performed. Total RNA was isolated from the heads of 30 flies (10 male or female flies per replication) using the reagent QIAzol Lysis Reagent (Qiagen, Hilden, Germany) according to the manufacturer’s protocol. The library was prepared according to the previously used protocol [24]. The double-stranded cDNA library was obtained using the NEBNext^®^ Ultra™ Directional RNA Library Prep kit for Illumina (NewEngland Biolabs, Ipswich, MA, USA) in accordance with the manufacturer’s protocol of 1 mg of total RNA. The number of libraries was determined via the qPCR method using the Rotor-Gene 6000 PCR system (Qiagen, Hilden, Germany) in accordance with the manufacturer’s protocol. The primers matched the sequences in the adapters, flanking the Illumina sequencing library. The insert size in cDNA libraries was approximately 260–270 bp. The quality of the libraries was determined using a highly sensitive DNA chip for the Agilent 2100 bioanalyzer (Agilent Technologies, Santa Clara, CA, USA) in accordance with the manufacturer’s protocol. Sequencing was carried out in single-end reads mode (76 bp) using the NextSeq500 System (Illumina, San Diego, CA, USA) using the equipment of EIMB RAS “Genome” center (https://www.eimb.ru/ru1/ckp/ccu_genome_ce.php, accessed on 18 May 2023).. At least 10 million reads were obtained for each sample. The obtained RNA-Seq are available in the NCBI Sequence Read Archive under the BioProject ID PRJNA929037.

### 4.3. Bioinformatic Analysis

Reads were checked up with FastQC 0.11.5, processed with trimmomatic 0.39 and mapped to the reference *D. melanogaster* genome BDGP6.32 (Ensembl release 107) with STAR 2.7.9a [81] and the reference GTF annotation. The 3′-bias was evaluated with RSeQC 3.0.1 [82]. Read counts per gene were calculated with the featureCounts tool from the subread 1.6.0 package.

To fight the differences in 3′-bias coming from the Trizol (QIAzol) method of RNA extraction, we used three correction methods (about them is written in detail below). Then, during further group comparisons for each gene, we chose the method out of the three that gave the minimum absolute value of the logarithm of expression fold change (LogFC). In the case of discrepancies between the results (differently directed expression fold changes), we considered LogFC = 0 for such a gene. Thus, this allowed us to increase confidence in the data: we state the fact of differential expression of a gene only if it is confirmed simultaneously by all 3 methods of 3′-bias correction.

Returning to the 3′-bias correction methods, the first of them consisted in counting the reads at the 3′-ends of transcripts, as suggested by Sigurgeirsson et al. [83]. The second correction method consisted in dividing the entire gene pool into 10 bins depending on average transcript length and re-normalizing the data by in each bin using TMM (edgeR). Additionally, a slope alignment was performed in the scatterplot drawn in Log[transcript length] versus LogFC coordinates (in particular, in the case of 3′-bias imbalance between groups, a strong correlation between transcript length and LogFC is usually observed). The third method consisted only in aligning the slope in the scatterplot.

The further processing was performed in the R 4.1.2 environment using the edgeR 3.36 package (differential expression) [84], clusterProfiler 4.2.2, ReactomePA 1.38, and topGO 2.46 packages (overrepresentation analysis with the KEGG, Gene Ontology, and Reactome databases) [85]. Before analysis, we filtered genes by expression level (CPM > 1.0) for at least 50% of the number of the samples in the smallest group.

### 4.4. Immunoblotting

Total protein extracts from flies’ heads were prepared using the ReadyPrep Protein Extraction Kit (Total Protein) (BioRad, Hercules, CA, USA) according to the manufacturer’s recommendations with addition of the Halt Protease and Phosphatase Inhibitor Cocktail (Thermo Scientific, Waltham, MA, USA). Thereafter, the protein samples were homogenized in SDS-PAGE buffer (65 mM Tris-HCl, pH 6.8; 10% glycerin; 2% SDS; 1% DTT; and 0.01% bromophenol blue) and heated to 100 °C for 5 min. Electrophoresis was performed in 8% PAAG, and the proteins were transferred to a Hybond ECL membrane (GE Healthcare, Chicago, IL, USA) using the semidry technique in buffer containing 25 mM Tris, 0.2 M glycine, 20% methanol, and 0.01% SDS with a current power of 1.5–2 mA/cm^2^ for 1.5 h. After the transfer, membranes were blocked using 5% ECL Blocking Agent (GE Healthcare, Chicago, IL, USA) in TBST (20 mM Tris-HCl, pH 7.6; 0.14 M NaCl; 0.01% Tween 20) for 1 h. After blocking, the membrane was incubated for 12 h with the following primary antibodies at the dilutions recommended by the manufacturers: anti-phospho-p44/42 MAPK (ERK1/2) (Thr202/Tyr204) polyclonal rabbit antibody (9101, Cell Signaling, Danvers, MA, USA) to detect phospho-ERK1/2. Next, the membrane was washed four times for 10 min each with TBST and incubated for 1 h with a horseradish peroxidase-conjugated secondary antibodies (anti-rabbit A0545 or anti-mouse A2304, depending on the types of primary antibodies used, Sigma-Aldrich, St Louis, MO, USA) at the dilutions recommended by the manufacturer. To remove bound primary and secondary antibodies and reprobe, the membranes were washed with Restore PLUS Western Blot Stripping Buffer (Thermo Fisher Scientific, Waltham, MA, USA). For signal detection, the SuperSignal West Femto PLUS Chemiluminescent Substrate (Thermo Fisher Scientific, Waltham, MA, USA) was used. The signal intensities were measured by means of the gel-documenting system ChemiDoc MP and the Quantity One 1-D Analysis Software program (BioRad, Hercules, CA, USA). The optical densities of the bands were analyzed in the quantized image by means of the ImageJ application.

### 4.5. Quantitative PCR

cDNA samples were obtained from the mRNA template using Mint reverse transcriptase and oligo(dT) primer (20 µM) according to the manufacturer’s protocol (Evrogen, Moscow, Russia). qPCR was performed in three technical replicates on an Applied Biosystems 7500 instrument (Thermo Fisher Scientific). We used three reference genes—*eIF4A*, *RpL32*, and *alphaTub84D*. The relative expressions (Torin-2 to control) of two target genes have been evaluated: *Srr* and *JhI-21*. First, ΔC_T_ (reference gene–target gene) values were calculated for *Srr* (or *JhI-21*) and then averaged within each age-sex group. Then, we calculated the difference of these average values between the Torin-2 and control groups (i.e., –ΔΔC_T_). The –ΔΔC_T_ values have been directly compared to the Log2FC values derived from RNA-Seq. The data were analyzed for each reference gene separately, and then for all three reference genes (by averaging ΔC_T_ across them). *alphaTub84D* showed itself to be not very stable and deviant from *eIF4A*, *RpL32*. In turn, *eIF4A* and *RpL32* genes showed similar results.

### 4.6. Statistical Analysis

To evaluate the statistical differences in survival curves, median lifespan, and maximum lifespan between control and experimental groups, the log-rank test, Fisher exact test, and Wang–Allison test [86] were used, respectively. Statistical analyses of the data were performed using R (The R Foundation), Microsoft Office Excel, and OASIS 2 (Online Application for Survival Analysis 2; Microsoft, Redmond, WA, USA) [87].

## 5. Conclusions

Our study investigated the role and potential mechanism of Torin-2′s effect on the *D. melanogaster* brain transcriptome. We have demonstrated that Torin-2 can slightly compensate for some age-dependent changes in the brain and increase the lifespan of males.

In particular, under the chronic administration of Torin-2, we observed signs of increased expression of heat shock proteins, gluconeogenesis genes, some genes regulating neuroplasticity and neurotransmission, cell adhesion, and intercellular contacts. On the other hand, we noticed decreased expression of genes participating in translation and mitochondrial function.

We hypothesize that Torin-2 may have effect on the glycine site of the glutamate receptor (NMDAR) Torin-2 may also have a significant positive effect on cell adhesion and active cytoskeleton, which play a significant role in synaptic contacts. Additionally, Torin-2 may also activate the MAPK pathway to protect against neurodegeneration. Torin-2 is able to modulate the activity of many cellular processes, and its complex effect may also be realized via interplay of the immune system, hormonal background, and metabolism. This question requires further research.

## Data Availability

The obtained RNA-Seq are available in the NCBI Sequence Read Archive under the BioProject ID PRJNA929037.

## Supplementary Materials

The following supporting information can be downloaded at: https://www.mdpi.com/article/10.3390/ijms24109095/s1, Figure S1: qPCR evaluation of gene expression changes under the chronic administration of Torin-2; Figure S2: Venn diagram which shows an overlap of the lists of genes whose expression changes significantly with age (*p* < 0.05) in control and Torin-fed flies of different genders; Table S1: Lifespan effects of Torin-2 treatment taken with various concentrations; Table S2: Differential gene expression data summary on pooled comparison of Torin-2 treated versus control group; Table S3: Differential gene expression data summary on various comparisons, only MAPK pathway.

## Abbreviations

TORC1/2	target of rapamycin complex1/complex2
MAPK	a mitogen-activated protein kinase
NMDA	the N-methyl-D-aspartate receptor
LTP	long-term potentiation
BBB	blood–brain barrier
ERK1/2 extracellular signal	regulated kinase 1/2
DEGs	differentially expressed genes
AMPs	antimicrobial peptides
cAMP	cyclic adenosine monophosphate
TCA	the citric acid cycle
