# Supplementary material for: Transcriptomic Analysis of the Effect of Torin-2 on the Central Nervous System of Drosophila melanogaster
Source: Int J Mol Sci. 2023 May 22;24(10):9095. doi: 10.3390/ijms24109095 (PMC10219255; doi:10.3390/ijms24109095)
Supplement: Supplementary file 1 [file ijms-24-09095-s001.zip › ijms-2227594-supplementary.pdf]

Supplementary Figure S1. qPCR evaluation of gene expression changes under the chronic administration of Torin-2. *eIF4A* is taken as the reference gene

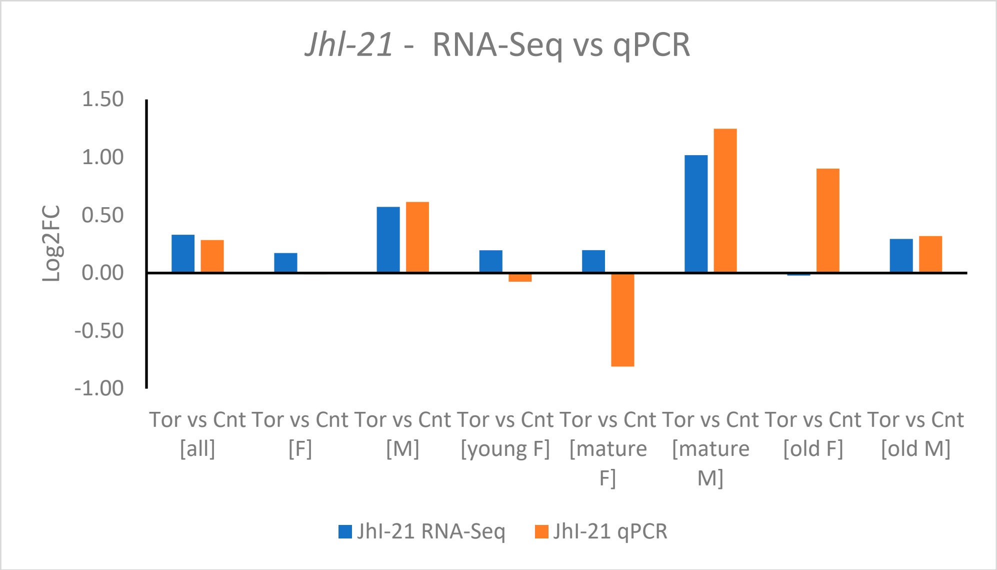

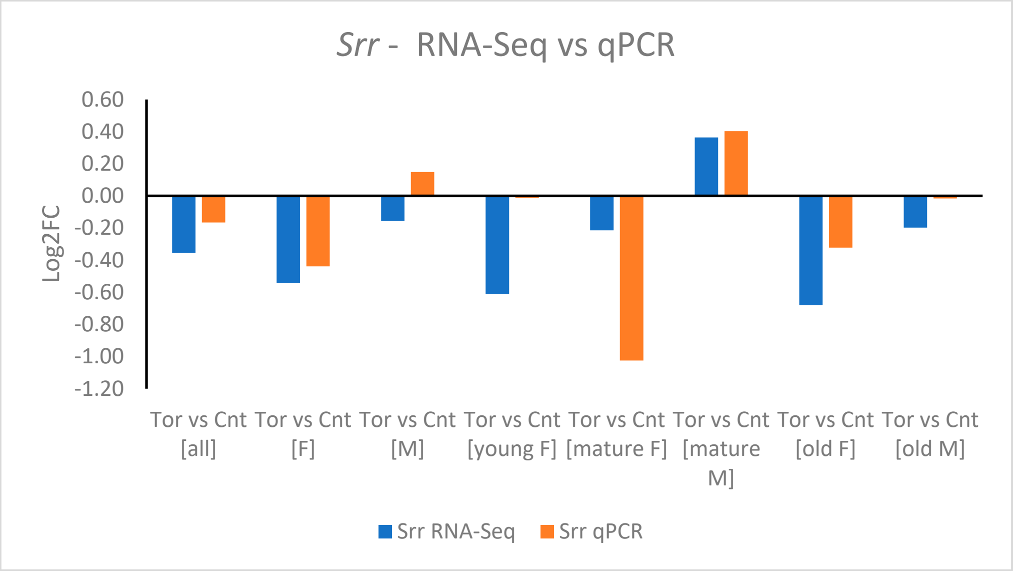

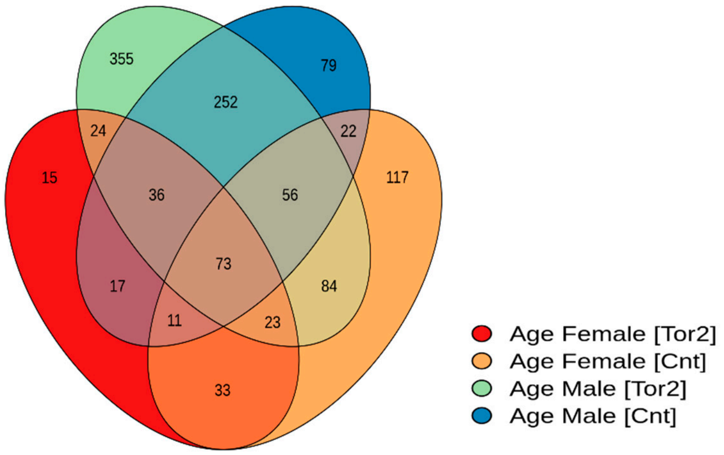

Supplementary Figure S2. Venn diagram, which shows an overlap of the lists of genes whose expression changes significantly with age ($p < 0.05$) in control and Torin-feeded flies of different genders.

Supplementary Table S1. Lifespan effects of Torin-2 treatment taken with various concentrations

	Median (days)	delta-Median (%)	Fisher's exact test		90th percentile (days)	delta 90th percentile (%)	Fisher's exact test		Wang-Allison test		N, flies
			p	FDR			p	FDR	p	FDR	
Males											
Control	46		n/a	n/a	55		n/a	n/a	n/a	n/a	442
0.5 µM	48	4	p<0.0001	p<0.0001	57	3.6	p>0.05	p>0.05	p>0.05	p>0.05	490
1 µM	48	4	p<0.0001	p<0.0001	57	3.6	p<0.05	p>0.05	p>0.05	p>0.05	484
5 µM	46	0	p>0.05	p>0.05	55	0	p>0.05	p>0.05	p>0.05	p>0.05	460
10 µM	46	0	p>0.05	p>0.05	56	1.8	p>0.05	p>0.05	p>0.05	p>0.05	507
Females											
Control	55	n/a	n/a	n/a	63	n/a	n/a	n/a	n/a	n/a	470
0.5 µM	55	0	p>0.05	p>0.05	64	1.6	p>0.05	p>0.05	p<0.05	p>0.05	495
1 µM	55	0	p>0.05	p>0.05	63	0	p>0.05	p>0.05	p>0.05	p>0.05	480
5 µM	54	-2	p>0.05	p>0.05	61	-3	p<0.05	p<0.05	p>0.05	p>0.05	494
10 µM	54	-2	p>0.05	p>0.05	63	0	p>0.05	p>0.05	p>0.05	p>0.05	474

Supplementary Table S2. Differential gene expression data on pooled comparison of Torin-2-treated versus Control groups. p-values were not FDR-adjusted. LogFC is taken as "minimal" across 3 methods of 3'-bias adjustment, p-value is taken as "maximal".

**Symbol**	**Log_2FC**	**p (QLF test)**	**Symbol**	**Log_2FC**	**p (QLF test)**
cort	2,03	0,0007	*Rassf*	0,2	0,02
Or42b	1,07	0,04	*Sirt2*	-0,16	0,05
Or1a	0,9	0,04	*Mondo*	-0,16	0,03
CG11529	0,67	0,007	*CG8858*	-0,17	0,03
E23	0,54	0,003	*CG8405*	-0,17	0,04
bip1	0,53	0,01	*hook*	-0,17	0,02
Prat	0,52	0,03	*CG11873*	-0,19	0,03
CG5290	0,5	0,01	*mRpS29*	-0,21	0,05
CG10466	0,44	0,01	*CG12531*	-0,22	0,03
Stip1	0,43	0,004	*Atx2*	-0,24	0,02
CG17816	0,4	0,01	*dbr*	-0,25	0,02
CG5380	0,38	0,02	*CG7997*	-0,26	0,04
Bdp1	0,38	0,006	*CG34354*	-0,26	0,04
RfC4	0,38	0,02	*Golgin104*	-0,28	0,03
SmD3	0,37	0,03	*CG10731*	-0,28	0,04
CG15270	0,37	0,04	*tn*	-0,29	0,02
BCL7-like	0,35	0,03	*CG7470*	-0,32	0,008
arx	0,33	0,04	*HERC2*	-0,35	0,006
GstE1	0,33	0,02	*Srr*	-0,35	0,01
CG16908	0,32	0,03	*eya*	-0,36	0,02
CG17378	0,32	0,02	*AMPKalpha*	-0,36	0,01
Sema2a	0,31	0,01	*CG32085*	-0,38	0,008
CG9667	0,29	0,04	*Ugt36E1*	-0,41	0,02
mbf1	0,25	0,02	*CG13896*	-0,45	0,05
Lsm10	-0,5	0,02			

Supplementary Table S3. Differential gene expression data summary on various comparisons, only MAPK pathway (GO:0000165). m1, m2, m3 – various methods of 3'-bias adjustment, m4 – not adjusted.

Gene ID	Symbol	Name	LogFC: Tor2 vs Cnt [Young, females]				LogFC: Tor2 vs Cnt [Adult, females]				LogFC: Tor2 vs Cnt [Old, females]			
			m1:3t-sl	*m2:bins-sl*	*m3:sl*	*m4:none*	*m1:3t-sl*	*m2:bins-sl*	*m3:sl*	*m4:none*	*m1:3t-sl*	*m2:bins-sl*	*m3:sl*	*m4:none*
FBgn0016756	Usp47	Ubiquitin specific protease 47	-0.50	-0.30	-0.26	-0.16	-0.36	-0.49	-0.53	-0.60	-0.44	-0.35	-0.30	-0.20
FBgn0001291	Jra	Jun-related antigen	-0.37	-0.10	-0.14	-0.18	0.62	0.56	0.57	0.57	-0.51	-0.32	-0.37	-0.39
FBgn0000711	flw	flapwing	0.94	0.34	0.32	0.34	-1.58	-0.28	-0.25	-0.28	1.19	0.25	0.21	0.25
FBgn0264493	rdx	roadkill	0.20	0.43	0.44	0.49	0.80	0.57	0.54	0.50	0.63	0.47	0.51	0.56
FBgn0014388	sty	sprouty	1.43	0.42	0.57	0.63	0.73	-0.27	-0.32	-0.37	0.28	0.14	0.25	0.31
FBgn0020767	Spred	Sprouty-related protein with EVH-1 domain	0.95	0.58	0.59	0.63	-0.57	-0.32	-0.34	-0.38	0.53	0.16	0.18	0.23
FBgn0024811	Crk	Crk oncogene	0.47	0.50	0.46	0.39	0.02	-0.33	-0.23	-0.21	0.25	0.41	0.39	0.34
FBgn0003371	sgg	shaggy	0.54	0.53	0.56	0.61	-0.45	-0.29	-0.30	-0.35	0.16	0.27	0.29	0.35
FBgn0015286	Rala	Ras-like protein A	0.04	0.37	0.37	0.38	0.41	0.34	0.37	0.34	0.08	0.08	0.07	0.09
FBgn0003209	raw	raw	0.61	0.41	0.49	0.55	-0.23	-0.43	-0.47	-0.52	0.07	-0.06	0.04	0.11
FBgn0032682	grnd	grindelwald	0.27	0.73	0.72	0.70	-0.22	-0.39	-0.26	-0.27	0.26	0.45	0.36	0.36
**FBgn0003256**	**rl**	**rolled**	**1.61**	**0.89**	**0.88**	**0.90**	**0.13**	**-0.22**	**-0.18**	**-0.21**	**0.53**	**0.36**	**0.32**	**0.35**
FBgn0086779	step	steppke	1.05	0.22	0.28	0.32	-1.28	-0.02	-0.06	-0.10	0.81	0.31	0.34	0.39
FBgn0036934	sNPF-R	short neuropeptide F receptor	0.44	0.54	0.69	0.76	1.44	0.34	0.25	0.20	1.88	1.11	1.22	1.29
FBgn0014006	Ask1	Apoptotic signal-regulating kinase 1	-0.29	-0.24	-0.20	-0.11	0.65	0.40	0.35	0.28	-0.45	-0.41	-0.36	-0.26
FBgn0038331	Ccm3	Cerebral cavernous malformation 3	0.88	0.35	0.31	0.27	-0.42	-0.08	-0.02	-0.02	0.44	0.26	0.20	0.19
FBgn0000173	ben	bendless	0.13	0.26	0.25	0.22	-0.07	-0.33	-0.19	-0.19	-0.10	0.12	0.06	0.05
FBgn0020224	Cbl	Cbl proto-oncogene	0.54	-0.10	-0.01	0.06	-0.88	-0.28	-0.34	-0.40	0.72	0.02	0.11	0.18
FBgn0033153	Gadd45	Growth arrest and DNA damage-inducible 45	0.03	0.61	0.54	0.51		-1.62	-1.61	-1.61	-0.27	-0.18	-0.22	-0.24
FBgn0005672	spi	spitz	0.49	0.30	0.25	0.22	0.25	0.40	0.45	0.44	0.06	0.11	0.05	0.04

Gene ID	Symbol	Name	LogFC: Tor2 vs Cnt [Young, males]				LogFC: Tor2 vs Cnt [Adult, males]				LogFC: Tor2 vs Cnt [Old, males]			
			m1:3t-sl	*m2:bins-sl*	*m3:sl*	*m4:none*	*m1:3t-sl*	*m2:bins-sl*	*m3:sl*	*m4:none*	*m1:3t-sl*	*m2:bins-sl*	*m3:sl*	*m4:none*
FBgn0016756	Usp47	Ubiquitin specific protease 47	0.48	0.34	0.35	0.40	-0.17	0.17	0.04	0.14	0.13	0.27	0.23	0.38
FBgn0001291	Jra	Jun-related antigen	0.30	0.38	0.36	0.36	0.65	0.54	0.70	0.68	0.18	0.09	0.08	0.05
FBgn0000711	flw	flapwing	-0.42	-0.17	-0.16	-0.14	-0.19	-0.51	-0.40	-0.37	0.27	0.01	0.08	0.13
FBgn0264493	rdx	roadkill	0.23	0.02	0.04	0.06	-0.52	-0.65	-0.62	-0.57	-0.02	-0.02	0.06	0.14
FBgn0014388	sty	sprouty	0.46	0.32	0.35	0.38	-0.58	-0.46	-0.56	-0.49	0.94	0.23	0.25	0.35
FBgn0020767	Spred	Sprouty-related protein with EVH-1 domain	-0.07	-0.02	0.01	0.03	-0.31	-0.50	-0.45	-0.41	0.22	0.29	0.38	0.46
FBgn0024811	Crk	Crk oncogene	-0.20	-0.04	-0.07	-0.09	-0.30	-0.39	-0.39	-0.44	0.21	0.25	0.19	0.11
FBgn0003371	sgg	shaggy	0.01	0.00	0.02	0.05	-0.47	-0.47	-0.43	-0.38	-0.07	0.13	0.20	0.29
FBgn0015286	Rala	Ras-like protein A	-0.64	-0.30	-0.29	-0.27	-0.56	-0.70	-0.57	-0.55	-0.07	-0.19	-0.09	-0.06
FBgn0003209	raw	raw	0.28	0.13	0.17	0.20	0.36	0.84	0.75	0.81	0.55	0.35	0.36	0.46
FBgn0032682	grnd	grindelwald	0.33	0.19	0.18	0.19	-0.40	-0.51	-0.31	-0.31	-0.03	-0.03	0.01	0.01
**FBgn0003256**	**rl**	**rolled**	**-0.31**	**-0.48**	**-0.47**	**-0.45**	**0.13**	**-0.30**	**-0.17**	**-0.15**	**0.32**	**0.04**	**0.12**	**0.16**
FBgn0086779	step	steppke	-0.59	-0.20	-0.18	-0.16	-0.24	-0.38	-0.34	-0.30	0.40	-0.03	0.06	0.14
FBgn0036934	sNPF-R	short neuropeptide F receptor		0.27	0.31	0.34		-0.50	-0.58	-0.52		0.47	0.48	0.59
FBgn0014006	Ask1	Apoptotic signal-regulating kinase 1	-0.18	-0.13	-0.12	-0.08	-0.05	0.11	-0.01	0.08	-0.17	-0.26	-0.28	-0.14
FBgn0038331	Ccm3	Cerebral cavernous malformation 3	-0.16	-0.24	-0.27	-0.27	-0.31	-0.53	-0.34	-0.36	0.29	0.11	0.10	0.08
FBgn0000173	ben	bendless	-0.25	-0.15	-0.15	-0.15	-0.47	-0.79	-0.59	-0.61	-0.11	-0.16	-0.10	-0.12
FBgn0020224	Cbl	Cbl proto-oncogene	0.01	0.23	0.25	0.29	-0.20	-0.32	-0.41	-0.34	0.38	0.15	0.17	0.27
FBgn0033153	Gadd45	Growth arrest and DNA damage-inducible 45	0.62	0.51	0.46	0.46	0.64	0.52	0.69	0.66	0.27	0.28	0.28	0.25
FBgn0005672	spi	spitz	0.25	0.20	0.18	0.17	0.51	0.27	0.44	0.42	-0.01	0.01	0.00	-0.02

FBgn0267698	Pak	p21-activated kinase	0.80	0.37	0.47	0.54	-0.48	-0.13	-0.18	-0.23	0.79	0.17	0.26	0.34	0.25	-0.13	-0.09	-0.06	0.21	-0.09	-0.18	-0.11	-0.30	0.17	0.18	0.29
FBgn0003079	Raf	Raf oncogene	0.07	-0.16	-0.08	-0.01	-0.21	0.21	0.14	0.09	0.03	-0.29	-0.20	-0.13	-0.27	-0.31	-0.29	-0.26	0.13	0.47	0.37	0.43	-0.53	-0.27	-0.25	-0.15
FBgn0086346	ALiX	ALG-2 interacting protein X	-0.12	-0.16	-0.15	-0.11	-0.28	-0.42	-0.48	-0.52	-0.69	-0.59	-0.55	-0.51	0.09	-0.02	0.00	0.02	0.45	0.47	0.53	0.57	0.22	0.32	0.40	0.47
FBgn0011230	poe	purity of essence	-0.70	-0.39	-0.47	-0.25	0.35	0.27	0.28	0.15	-0.35	-0.37	-0.41	-0.20	-0.07	-0.09	-0.14	-0.05	0.13	0.24	0.03	0.22	-0.16	-0.08	-0.27	0.05
FBgn0086378	Alg-2	Apoptosis-linked gene-2	0.79	1.53	1.46	1.41	-0.15	-0.33	-0.22	-0.21	0.25	0.30	0.26	0.22	0.11	-0.20	-0.25	-0.26	-0.33	-0.45	-0.46	-0.50	0.06	0.04	-0.04	-0.10
FBgn0020249	stck	steamer duck	-0.31	-0.26	-0.29	-0.33	0.08	0.07	0.10	0.11	0.08	0.15	0.09	0.06	-0.24	-0.27	-0.29	-0.30	0.27	0.14	0.33	0.30	0.01	-0.12	-0.11	-0.15
FBgn0032210	CYLD	Cylindromatosis	0.16	0.32	0.29	0.29	0.12	-0.19	-0.04	-0.06	0.27	0.40	0.32	0.33	-0.06	-0.16	-0.17	-0.16	-0.01	-0.38	-0.19	-0.18	-0.17	-0.19	-0.16	-0.15
FBgn0004638	drk	downstream of receptor kinase	0.86	0.35	0.34	0.34	-0.26	-0.23	-0.19	-0.21	0.35	0.12	0.09	0.11	-0.29	-0.04	-0.03	-0.02	-0.25	-0.75	-0.62	-0.61	0.06	-0.22	-0.13	-0.11
FBgn0040281	Aplip1	APP-like protein interacting protein 1	0.60	0.30	0.24	0.25	-0.35	-0.50	-0.51	-0.54	0.34	0.42	0.37	0.39	-0.09	0.15	0.17	0.18	-0.11	-0.21	-0.08	-0.06	-0.07	0.07	0.15	0.19
FBgn0040068	Vav	Vav guanine nucleotide exchange factor	0.86	0.23	0.30	0.37	-0.81	-0.04	-0.08	-0.14	0.66	0.22	0.30	0.37	-0.15	-0.11	-0.08	-0.05	-0.21	-0.14	-0.24	-0.17	0.04	0.13	0.14	0.25
FBgn0024846	p38b	p38b MAP kinase	-0.14	-0.06	-0.09	-0.10	-1.54	-0.94	-0.96	-0.97	-0.44	-0.57	-0.63	-0.63	0.10	-0.10	-0.10	-0.10	1.40	0.68	0.83	0.82	-0.13	0.21	0.26	0.25
FBgn0010269	Dsor1	Downstream of raf1	-0.54	-0.55	-0.61	-0.64	-0.54	-0.32	-0.33	-0.34	-0.14	0.17	0.09	0.09	0.35	0.54	0.51	0.51	0.64	0.15	0.31	0.30	0.46	0.66	0.66	0.64
FBgn0283500	Sac1	Sac1 phosphatase	-0.05	-0.25	-0.27	-0.25	-0.96	-0.67	-0.71	-0.73	0.04	-0.18	-0.22	-0.19	0.05	0.21	0.23	0.25	-0.25	-0.09	0.04	0.06	0.19	0.17	0.24	0.27
FBgn0035601	Uev1A	Ubiquitin-conjugating enzyme variant 1A	0.20	0.24	0.22	0.17	-0.17	-0.14	-0.07	-0.06	0.16	0.09	0.03	0.00	-0.05	-0.10	-0.11	-0.12	-0.43	-0.73	-0.52	-0.56	-0.09	-0.21	-0.20	-0.25
FBgn0035688	fmt	fiery mountain	0.08	0.13	0.18	0.28	-0.26	-0.09	-0.14	-0.21	0.45	0.14	0.20	0.30	-0.20	-0.11	-0.10	-0.06	-0.19	-0.24	-0.36	-0.26	-0.22	-0.08	-0.11	0.04
FBgn0086677	jeb	jelly belly	0.27	-0.07	-0.03	0.11	-0.28	-0.13	-0.17	-0.26	-0.11	-0.04	-0.02	0.12	-0.13	-0.14	0.14	-0.08	-0.67	-0.71	-0.85	-0.73	-0.40	-0.23	-0.30	-0.09
FBgn0036844	Mkp3	Mitogen-activated protein kinase phosphatase 3	0.20	0.16	0.18	0.22	-0.33	-0.33	-0.36	-0.40	-0.10	-0.02	0.01	0.06	-0.87	-0.21	-0.19	-0.17	-0.15	-0.36	-0.32	-0.28	-0.19	0.14	0.22	0.30
FBgn0260635	Diap1	Death-associated inhibitor of apoptosis 1	0.10	0.13	0.09	0.09	-0.26	-0.51	-0.50	-0.52	-0.04	0.06	-0.02	-0.01	0.26	0.34	0.34	0.34	0.28	0.07	0.25	0.25	0.79	0.73	0.75	0.76
FBgn0036756	Cln3	Cln3	0.38	0.30	0.26	0.23	0.47	0.06	0.12	0.12	-0.47	-0.23	-0.32	-0.34	-0.19	-0.22	-0.24	-0.24	0.36	-0.15	0.03	0.01	-0.50	-0.37	-0.38	-0.40
FBgn0015513	mbc	myoblast city	1.57	0.02	0.04	0.17	-1.38	0.26	0.22	0.13	0.92	0.07	0.10	0.24	-0.54	-0.26	-0.28	-0.22	-0.18	0.08	-0.07	0.05	0.32	-0.12	-0.20	0.01
FBgn0036932	CG14184		-0.15	-0.17	-0.19	-0.31	0.14	0.27	0.15	0.19	0.12	0.15	0.15	0.06	0.05	-0.03	-0.04	-0.08	-0.18	0.03	-0.27	-0.37	-0.20	-0.19	-0.30	-0.45
FBgn0267487	Ptp61F	Protein tyrosine phosphatase 61F	0.73	0.45	0.49	0.52	0.30	-0.06	-0.09	-0.12	0.16	0.19	0.23	0.27	0.14	0.12	0.14	0.16	0.03	0.06	0.12	0.15	-0.16	0.01	0.12	0.18
FBgn0263395	hppy	happyhour	1.64	0.36	0.42	0.50	-0.68	0.07	0.03	-0.03	0.48	0.13	0.21	0.30	-0.43	-0.17	-0.16	-0.12	0.50	0.27	0.16	0.24	0.01	0.05	0.04	0.17
FBgn0001234	lncRNA:Hsromega	Heat shock RNA omega	-0.35	-0.29	-0.26	-0.12	0.16	-0.05	-0.07	-0.16	0.01	0.24	0.25	0.39	0.10	0.24	0.22	0.28	-0.23	-0.19	-0.36	-0.22	0.00	-0.02	-0.10	0.12
FBgn0004436	Ubc6	Ubiquitin conjugating enzyme 6	0.57	0.40	0.35	0.32	-0.02	-0.20	-0.12	-0.13	0.17	0.18	0.10	0.09	-0.01	0.20	0.17	0.17	-0.13	-0.55	-0.36	-0.38	-0.01	0.05	0.03	0.01

FBgn0015765	p38a	p38a MAP kinase	-0.01	0.21	0.15	0.12	-0.36	-0.38	-0.30	-0.30	-0.29	-0.07	-0.13	-0.15	0.08	0.05	0.04	0.03	-0.53	-0.67	-0.48	-0.51	-0.20	-0.15	-0.16	-0.19
FBgn0044323	Cka	Connector of kinase to AP-1	0.52	0.11	0.22	0.28	-0.70	-0.10	-0.15	-0.20	0.44	0.11	0.20	0.26	0.01	-0.09	-0.07	-0.04	-0.31	-0.37	-0.45	-0.39	0.11	-0.04	-0.01	0.09
FBgn0003731	Egfr	Epidermal growth factor receptor	0.26	-0.06	-0.03	0.08	-0.24	-0.30	-0.34	-0.42	-0.22	-0.24	-0.19	-0.08	-0.42	-0.36	-0.35	-0.30	-0.10	0.05	-0.08	0.02	-0.12	0.05	0.00	0.17
FBgn0032816	Nf-YB	Nuclear factor Y-box B	-0.31	-0.20	-0.25	-0.35	0.02	-0.20	-0.19	-0.16	0.32	0.28	0.33	0.25	0.17	0.08	0.08	0.05	-0.52	-0.40	-0.56	-0.64	0.05	0.01	-0.05	-0.17
FBgn0283499	InR	Insulin-like receptor	0.99	0.40	0.44	0.61	-0.02	0.18	0.14	0.03	0.16	0.38	0.39	0.55	0.03	0.02	0.00	0.08	-0.66	-0.62	-0.77	-0.62	0.19	0.07	-0.05	0.21
FBgn0003053	peb	pebbled	0.47	0.76	0.76	0.90		-0.95	-0.99	-1.07	0.58	0.13	0.16	0.30	-0.14	0.04	0.04	0.09	-0.34	-0.29	-0.44	-0.32	-0.10	-0.54	-0.61	-0.42
FBgn0265193	Atf-2	Activating transcription factor-2	-0.76	-0.39	-0.44	-0.49	0.21	-0.04	-0.03	-0.03	-0.08	0.02	-0.03	-0.06	0.11	0.08	0.07	0.06	0.63	0.71	0.88	0.85	-0.75	-0.64	-0.62	-0.68
FBgn0024326	Mkk4	MAP kinase kinase 4	0.12	0.30	0.32	0.36	-0.06	-0.06	-0.08	-0.12	-0.13	0.04	0.07	0.12	0.01	-0.08	-0.06	-0.03	-0.59	-0.43	-0.40	-0.35	-0.27	-0.26	-0.16	-0.08
FBgn0038167	Lkb1	Lkb1 kinase	-0.30	-0.64	-0.68	-0.67		0.27	0.24	0.21	0.14	0.19	0.14	0.18	1.06	0.46	0.47	0.49	0.71	0.55	0.68	0.70	0.26	0.28	0.35	0.39
FBgn0243512	puc	puckered	0.34	0.29	0.27	0.29	-0.50	-0.26	-0.26	-0.30	0.51	0.47	0.43	0.46	0.07	0.09	0.11	0.12	-0.17	-0.10	0.02	0.04	-0.16	-0.09	-0.03	0.02
FBgn0039532	Mtl	Mig-2-like	0.69	0.43	0.43	0.41	0.21	0.17	0.31	0.30	0.22	0.19	0.15	0.15	0.16	-0.11	-0.11	-0.11	0.18	0.09	0.28	0.27	-0.06	-0.09	-0.04	-0.05
FBgn0264324	spg	sponge	0.55	-0.20	-0.18	-0.04	0.98	0.43	0.40	0.31	1.22	-0.22	-0.18	-0.05	-0.41	-0.04	-0.05	0.01	-0.66	0.34	0.19	0.32	0.72	0.11	0.03	0.24
FBgn0010909	msn	misshapen	0.97	0.54	0.59	0.69	-0.18	-0.16	-0.22	-0.28	0.09	0.14	0.20	0.30	0.04	-0.04	-0.02	0.02	-0.07	-0.13	-0.25	-0.16	0.08	-0.09	-0.11	0.04
FBgn0031044	MKP-4	MAPK Phosphatase 4	0.23	-0.58	-0.60	-0.60		-0.44	-0.37	-0.39	0.21	0.57	0.50	0.51		0.35	0.35	0.35	-0.29	-0.48	-0.28	-0.28	0.26	0.27	0.29	0.31
FBgn0035437	Strip	Striatin interacting protein	-0.56	-0.36	-0.35	-0.29	-0.38	-0.08	-0.14	-0.19	0.08	-0.12	-0.10	-0.03	-0.21	-0.18	-0.16	-0.13	0.21	0.52	0.55	0.61	0.09	0.17	0.24	0.33
FBgn0032006	Pvr	PDGF- and VEGF-receptor related	-0.30	-0.04	0.01	0.11	-0.01	-0.49	-0.52	-0.59	0.02	-0.39	-0.32	-0.22	-0.32	-0.07	-0.06	-0.01	0.19	0.69	0.57	0.66	0.19	0.27	0.22	0.38
FBgn0050476	ave	aveugle	-0.21	-0.33	-0.32	-0.47	0.14	0.11	0.01	0.06	0.25	0.32	0.37	0.25	0.42	0.23	0.24	0.19	-0.38	-0.10	-0.38	-0.50	0.06	0.16	0.07	-0.12
FBgn0261456	hpo	hippo	1.30	0.30	0.28	0.30	-0.48	-0.02	-0.01	-0.04	1.28	0.20	0.15	0.18	-0.40	-0.19	-0.18	-0.17	-0.45	-0.21	-0.09	-0.06	0.28	0.15	0.23	0.28
FBgn0266377	Pde8	Phosphodiesterase 8	0.58	0.09	0.15	0.24	-1.10	-0.39	-0.45	-0.51	-0.48	-0.01	0.06	0.15	-0.09	0.17	0.19	0.23	0.04	0.03	-0.08	0.00	-0.20	0.09	0.08	0.21
FBgn0030976	CG7378		0.42	0.20	0.20	0.21	-0.36	-0.24	-0.26	-0.28	0.06	-0.02	-0.05	-0.03	0.16	0.16	0.17	0.19	-0.09	-0.02	0.10	0.11	0.15	-0.14	-0.05	-0.01
FBgn0032208	Ufd4	Ubiquitin fusion-degradation 4-like	-0.34	-0.12	-0.13	0.02	0.23	0.07	0.03	-0.06	-0.21	-0.15	-0.14	0.01	-0.38	-0.17	-0.19	-0.12	0.09	0.12	-0.04	0.09	-0.18	-0.15	-0.24	-0.02
FBgn0031885	Mnn1	Menin 1	0.02	-0.09	0.00	0.07	-0.02	-0.20	-0.28	-0.33	0.14	0.12	0.22	0.29	-0.01	-0.13	-0.11	-0.07	-0.60	-0.51	-0.61	-0.54	0.02	-0.08	-0.06	0.05
FBgn0034110	Atg9	Autophagy-related 9	-0.47	-0.28	-0.27	-0.25	-0.55	-0.71	-0.76	-0.79	-0.08	-0.06	-0.03	0.02	0.20	0.39	0.42	0.44	0.35	0.11	0.17	0.21	0.74	0.66	0.73	0.80
FBgn0033160	Dhx15	DEAH-box helicase 15	-0.51	-0.51	-0.52	-0.50	0.17	0.01	0.00	-0.04	0.20	0.07	0.02	0.06	0.45	0.36	0.37	0.39	0.02	0.14	0.26	0.29	-0.07	0.02	0.08	0.13
FBgn0267339	p38c	p38c MAP kinase	0.94	0.98	1.01	0.94	0.06	-0.56	-0.42	-0.42	-0.57	-0.29	-0.29	-0.34		-0.21	-0.30	-0.30	-0.15	-0.46	-0.43	-0.48	0.37	0.53	0.45	0.37

FBgn0037655	Kcmf1	Potassium channel modulatory factor 1	-0.55	-0.44	-0.49	-0.47	0.03	-0.04	-0.06	-0.08	0.05	0.10	0.06	0.09	0.21	0.20	0.21	0.23	0.08	-0.08	0.02	0.05	-0.22	-0.22	-0.15	-0.11
FBgn0030613	Rab3-GEF	Rab3 GDP-GTP exchange factor	-0.33	-0.29	-0.32	-0.17	-0.43	-0.29	-0.31	-0.41	-0.28	-0.16	-0.14	0.00	0.06	0.13	0.14	0.20	-0.08	-0.11	-0.26	-0.13	-0.29	-0.09	-0.18	0.04
FBgn0010303	hep	hemipterous	0.28	-0.05	0.01	0.10	-0.40	-0.55	-0.61	-0.68	0.50	0.22	0.29	0.38	-0.49	-0.23	-0.22	-0.18	0.42	0.31	0.20	0.29	-0.26	0.15	0.13	0.27
FBgn0034894	sigmar	salivary glands marred	1.01	0.06	0.03	0.02	-0.47	-0.27	-0.14	-0.15	0.38	0.47	0.40	0.41	0.37	0.01	0.01	0.01	-0.27	-0.40	-0.21	-0.22	-0.05	-0.01	0.02	0.02
FBgn0040206	krz	kurtz	1.78	0.38	0.50	0.55	-0.93	0.03	-0.04	-0.09	0.45	0.06	0.14	0.21	0.44	-0.04	-0.02	0.00	-0.01	0.10	0.02	0.07	0.19	-0.26	-0.24	-0.14
FBgn0035538	DopEcR	Dopamine/Ecdysteroid receptor	0.16	0.25	0.24	0.30	-0.04	0.11	0.10	0.05	-0.05	0.08	0.10	0.16	-0.27	-0.13	-0.11	-0.09	0.12	-0.01	0.02	0.07	-0.22	-0.22	-0.14	-0.05
FBgn0264922	smt3	smt3	0.17	0.28	0.23	0.12	-0.08	-0.02	-0.14	-0.10	0.06	0.14	0.16	0.07	0.02	0.06	0.05	0.01	-0.58	-0.32	-0.63	-0.72	0.04	0.14	0.03	-0.11
FBgn0032840	sNPF	short neuropeptide F precursor	0.29	0.20	0.18	0.19	-0.62	-0.12	-0.15	-0.18	0.98	0.29	0.26	0.28	0.27	0.01	0.02	0.03	-0.13	-0.06	0.06	0.09	0.67	0.03	0.11	0.15
FBgn0040505	Alk	Anaplastic lymphoma kinase	-0.16	0.00	0.00	0.15	0.38	-0.15	-0.18	-0.28	-0.01	0.12	0.13	0.28	0.07	0.02	0.00	0.07	-0.71	-0.63	-0.79	-0.64	-0.01	-0.03	-0.13	0.11
FBgn0034199	Gbp1	Growth-blocking peptide 1	0.01	-0.17	-0.14	-0.31	-0.20	0.06	-0.12	-0.05	-0.02	-0.07	0.00	-0.14	-0.02	-0.06	-0.05	-0.11	-0.41	-0.24	-0.51	-0.65	-0.12	-0.04	-0.09	-0.30
FBgn0026319	Traf4	TNF-receptor-associated factor 4		0.30	0.30	0.30		-0.53	-0.56	-0.59	0.84	0.48	0.45	0.48		0.86	0.87	0.88	0.84	0.02	0.09	0.12	0.43	0.67	0.73	0.77
FBgn0011706	rpr	reaper	-0.40	0.04	-0.06	-0.15	0.14	0.16	0.21	0.24	0.05	0.28	0.30	0.23	0.66	0.69	0.72	0.70	-0.57	-0.65	-0.79	-0.87	0.26	0.25	0.13	0.02
FBgn0259212	cno	canoe	0.53	0.14	0.17	0.31	-0.10	0.15	0.10	0.01	0.25	0.07	0.09	0.22	-0.46	-0.10	-0.11	-0.05	0.00	-0.08	-0.23	-0.10	0.33	0.02	-0.05	0.16
FBgn0024329	Mekk1	Mekk1	-0.64	-0.41	-0.39	-0.28	0.34	0.04	-0.01	-0.08	-0.02	-0.21	-0.17	-0.06	0.00	-0.16	-0.14	-0.10	0.27	0.70	0.58	0.67	0.08	0.21	0.17	0.32
FBgn0000370	crc	cryptocephal		0.48	0.49	0.49		-0.18	-0.14	-0.16		-0.19	-0.22	-0.20		-0.30	-0.28	-0.27		-0.45	-0.31	-0.30		-0.23	-0.12	-0.10
FBgn0261524	lic	licorne	0.05	0.16	0.13	0.13	0.36	0.34	0.41	0.39	0.01	-0.09	-0.14	-0.13	0.08	-0.05	-0.06	-0.05	0.30	0.40	0.58	0.58	-0.21	-0.06	-0.03	-0.03
FBgn0000229	bsk	basket	-0.08	0.04	-0.01	-0.04	0.17	0.02	0.10	0.09	0.05	0.11	0.04	0.03	-0.30	-0.20	-0.22	-0.23	-0.40	-0.62	-0.44	-0.45	0.04	-0.05	-0.06	-0.08
FBgn0004569	aos	argos	0.65	0.17	0.21	0.24	-0.33	0.12	0.08	0.05	-0.21	-0.12	-0.10	-0.05	0.41	0.34	0.34	0.36	-0.32	-0.03	0.01	0.05	0.13	0.09	0.19	0.25
FBgn0286070	cnk	connector enhancer of ksr	-0.48	-0.39	-0.35	-0.24	1.18	0.27	0.22	0.15	0.03	-0.14	-0.09	0.02	0.18	0.19	0.21	0.25	-0.75	0.22	0.09	0.19	0.17	0.09	0.04	0.20
FBgn0011573	Cdc37	Cdc37	-0.05	0.18	0.11	0.08	0.36	0.35	0.33	0.33	-0.31	-0.15	-0.21	-0.22	0.10	0.11	0.09	0.09	1.11	0.93	1.10	1.08	0.02	0.02	0.01	-0.01
FBgn0010333	Rac1	Rac1	0.49	0.17	0.15	0.13	-0.38	-0.15	-0.09	-0.10	-0.19	-0.03	-0.08	-0.09	-0.19	0.07	0.07	0.07	-0.20	0.14	0.32	0.31	0.34	-0.02	0.02	0.01
FBgn0016641	PTP-ER	Protein tyrosine phosphatase-ERK/Enhancer of Ras1	-0.23	-0.32	-0.27	-0.17	-0.16	-0.32	-0.36	-0.43	-0.01	-0.13	-0.07	0.03	-0.13	-0.05	-0.04	0.00	-0.45	-0.17	-0.28	-0.19	0.00	0.14	0.11	0.26
FBgn0031030	Tao	Tao	-0.48	0.00	0.00	0.04	0.63	0.42	0.39	0.35	0.03	0.16	0.19	0.24	-0.14	-0.02	-0.02	0.01	-0.24	-0.16	-0.13	-0.08	-0.05	-0.03	0.05	0.14
FBgn0034577	cpa	capping protein alpha	0.54	-0.03	-0.06	-0.07	0.08	0.27	0.33	0.32	-0.08	0.06	0.00	0.00	-0.23	0.06	0.05	0.06	0.18	0.45	0.62	0.62	0.04	-0.11	-0.07	-0.07
FBgn0010341	Cdc42	Cdc42	-0.16	-0.05	-0.07	-0.08	0.45	0.19	0.31	0.30	-0.20	-0.12	-0.16	-0.16	-0.17	-0.06	-0.06	-0.06	-0.03	-0.16	0.01	0.01	0.03	-0.12	-0.08	-0.08

FBgn0040078	pont	pontin	0.01	0.10	0.04	0.01	0.68	0.84	0.85	0.85	0.22	-0.04	-0.08	-0.09	0.13	-0.02	-0.05	-0.05	0.34	0.22	0.37	0.36	-0.16	-0.24	-0.25	-0.27
FBgn0030964	Pvf1	PDGF- and VEGF-related factor 1	-0.37	-0.21	-0.21	-0.21	-0.10	-0.42	-0.40	-0.43	0.14	0.25	0.25	0.26	-0.10	-0.19	-0.18	-0.18	0.58	0.20	0.33	0.33	0.37	0.18	0.26	0.28
FBgn0030674	HUWE1	HECT, UBA and WWE domain containing E3 ubiquitin protein ligase 1	0.11	-0.18	-0.26	-0.04	0.33	0.12	0.13	0.00	0.20	-0.06	-0.11	0.10	0.03	0.20	0.16	0.25	0.11	0.14	-0.07	0.13	-0.26	0.00	-0.19	0.13
FBgn0024187	syd	sunday driver	1.76	0.34	0.37	0.46	-0.10	0.13	0.08	0.02	0.52	0.01	0.08	0.17	-0.23	0.00	0.02	0.06	-0.09	0.10	-0.01	0.08	0.26	0.49	0.47	0.61
FBgn0028546	ics	icarus	-0.27	-0.06	-0.09	-0.14	0.17	0.11	0.18	0.18	0.08	0.09	0.03	0.01	0.18	0.04	0.03	0.02	-0.11	-0.32	-0.12	-0.16	-0.22	-0.31	-0.30	-0.34
FBgn0032409	Ced-12	Ced-12	-0.08	-0.05	-0.08	-0.05	-0.05	0.22	0.25	0.22	-0.16	-0.06	-0.10	-0.07	-0.02	-0.17	-0.16	-0.15	-0.31	-0.37	-0.25	-0.22	-0.18	-0.18	-0.10	-0.05
FBgn0004636	Rap1	Rap1 GTPase	0.10	-0.13	-0.07	-0.04	-0.03	0.11	0.08	0.04	-0.08	-0.16	-0.11	-0.07	-0.03	-0.22	-0.21	-0.19	-0.33	-0.38	-0.33	-0.30	-0.03	-0.09	0.02	0.08
FBgn0014011	Rac2	Rac2	-0.34	-0.05	-0.07	-0.08	0.20	-0.30	-0.15	-0.16	0.05	0.01	-0.05	-0.05	0.22	0.07	0.07	0.07	-0.47	-0.68	-0.49	-0.50	-0.07	-0.07	-0.03	-0.03
FBgn0003502	Btk29A	Btk family kinase at 29A	-0.52	0.10	0.18	0.25	0.18	0.16	0.09	0.04	0.12	0.17	0.24	0.31	-0.30	-0.17	-0.16	-0.13	-0.07	-0.12	-0.22	-0.15	-0.28	0.01	0.03	0.13
FBgn0011570	cpb	capping protein beta	-0.11	0.06	0.00	-0.02	-0.38	0.15	0.17	0.17	-0.32	-0.15	-0.21	-0.22	0.14	0.02	0.00	0.00	0.34	0.23	0.40	0.38	-0.09	-0.10	-0.12	-0.14
FBgn0003205	Ras85D	Ras oncogene at 85D	1.51	0.25	0.23	0.22	-0.41	0.03	0.17	0.15	0.59	0.12	0.05	0.06	0.25	0.06	0.05	0.05	0.15	-0.19	0.00	0.00	0.40	-0.09	-0.06	-0.06
FBgn0010407	Ror	Ror	-0.32	-0.02	-0.07	-0.06	0.35	-0.03	-0.01	-0.03	-0.07	0.30	0.27	0.29	0.11	0.11	0.13	0.14	-0.07	-0.22	-0.10	-0.09	-0.46	-0.37	-0.27	-0.24
FBgn0025743	mbt	mushroom bodies tiny	-0.46	-0.09	-0.06	-0.01	0.48	0.12	0.08	0.03	-0.14	0.02	0.05	0.11	0.10	0.41	0.42	0.45	-0.07	-0.01	0.02	0.07	0.43	0.43	0.52	0.60
FBgn0086361	alph	alphabet	-0.50	-0.13	-0.14	-0.13	0.25	0.26	0.28	0.25	-0.04	0.08	0.05	0.07	-0.31	0.05	0.06	0.07	-0.15	-0.17	-0.05	-0.03	-0.12	-0.04	0.05	0.08
FBgn0003139	PpV	Protein phosphatase V	-0.07	0.02	-0.02	-0.05	0.56	0.36	0.43	0.42	0.11	0.12	0.06	0.05	0.20	-0.04	-0.07	-0.07	0.09	-0.06	0.13	0.11	-0.14	-0.10	-0.11	-0.13
FBgn0261477	slim	scruin like at the midline	0.28	-0.09	-0.05	-0.02	-0.38	-0.09	-0.14	-0.18	0.17	-0.12	-0.09	-0.04	0.20	0.07	0.09	0.11	0.08	0.07	0.11	0.15	0.06	-0.16	-0.05	0.00
FBgn0013987	MAPk-Ak2	MAP kinase activated protein-kinase-2	-0.12	-0.04	-0.07	-0.07	0.21	0.16	0.22	0.20	0.05	0.11	0.03	0.04	-0.14	-0.02	-0.02	-0.02	0.22	0.29	0.46	0.46	0.20	0.01	0.03	0.04
FBgn0026323	Tak1	TGF-beta activated kinase 1	-0.54	-0.21	-0.23	-0.22	-0.20	-0.13	-0.17	-0.20	0.01	-0.05	-0.07	-0.04	-0.26	-0.04	-0.03	-0.02	0.36	0.15	0.26	0.29	-0.32	-0.02	0.05	0.09
FBgn0037621	M1BP	Motif 1 Binding Protein	-0.07	-0.01	-0.09	-0.13	0.28	0.65	0.68	0.68	0.02	0.17	0.11	0.09	-0.01	-0.02	-0.04	-0.05	0.23	0.37	0.54	0.52	-0.09	-0.02	-0.02	-0.05
FBgn0028717	Lnk	Lnk	-0.36	-0.20	-0.23	-0.18	-0.02	0.02	-0.01	-0.06	0.13	0.27	0.30	0.35	0.26	0.27	0.30	0.32	0.00	-0.09	-0.05	-0.01	-0.02	0.33	0.41	0.48
FBgn0025637	SkpA	SKP1-related A	0.01	0.09	0.07	-0.01	0.08	-0.05	0.06	0.08	-0.01	0.12	0.10	0.05	0.14	0.01	-0.01	-0.03	-0.49	-0.50	-0.48	-0.54	-0.09	-0.06	-0.11	-0.20
FBgn0038972	CG7054		0.11	0.03	-0.03	-0.12	0.15	0.05	0.07	0.10	-0.03	0.02	0.05	-0.02	-0.02	-0.07	-0.07	-0.10	-0.32	-0.24	-0.41	-0.48	-0.12	-0.05	-0.14	-0.25
FBgn0037327	PEK	pancreatic eIF-2alpha kinase	0.21	-0.20	-0.13	-0.05	0.24	0.04	0.00	-0.06	-0.01	-0.23	-0.15	-0.07	-0.15	0.15	0.16	0.20	0.02	0.29	0.18	0.25	-0.10	0.07	0.06	0.19
FBgn0264959	Src42A	Src oncogene at 42A	1.42	-0.13	-0.07	0.02	0.18	0.29	0.24	0.17	0.73	-0.02	0.05	0.14	-0.12	-0.03	-0.03	0.01	-0.06	0.02	-0.09	-0.01	-0.22	-0.09	-0.11	0.03
FBgn0015295	Shark	SH2 ankyrin repeat kinase	0.10	-0.17	-0.14	-0.11	0.48	0.27	0.20	0.16	-0.42	-0.22	-0.24	-0.18	0.10	0.43	0.49	0.50	0.07	0.25	0.29	0.34	0.25	0.40	0.47	0.55

FBgn0053276	Urm1	Ubiquitin-related modifier 1	0.73	0.12	0.19	0.01	-0.13	-0.02	-0.12	-0.05	0.15	0.12	0.15	0.00	0.12	0.13	0.12	0.06	-0.62	-0.34	-0.60	-0.76	0.59	0.34	0.28	0.06
FBgn0265778	PDZ-GEF	PDZ domain-containing guanine nucleotide exchange factor		0.49	0.53	0.65		-0.26	-0.28	-0.36		0.27	0.32	0.43		-0.11	-0.11	-0.06		0.27	0.13	0.24		0.16	0.11	0.28
FBgn0263933	ebi	ebi	-0.06	-0.09	-0.08	-0.02	0.21	-0.03	-0.08	-0.12	0.08	-0.01	0.03	0.08	0.11	-0.12	-0.11	-0.08	-0.16	0.04	0.08	0.13	0.43	0.07	0.15	0.24
FBgn0039859	CG11539		-0.33	-0.45	-0.48	-0.58	-0.20	-0.03	-0.19	-0.15	-0.09	-0.14	-0.14	-0.22	-0.33	-0.05	-0.06	-0.11	-0.03	0.07	-0.20	-0.29	0.02	-0.07	-0.22	-0.35
FBgn0039338	XNP	XNP	0.16	-0.03	0.03	0.11	-0.23	-0.06	-0.09	-0.16	0.14	-0.16	-0.09	0.00	0.06	0.06	0.07	0.11	0.08	0.26	0.16	0.23	-0.06	0.24	0.22	0.35
FBgn0003984	vn	vein	-0.25	0.05	0.17	0.25	-0.02	-0.26	-0.29	-0.35	0.21	0.09	0.15	0.24	-0.15	0.08	0.09	0.12	-0.48	-0.34	-0.44	-0.35	-0.01	-0.03	-0.05	0.08
FBgn0265464	Traf6	TNF-receptor-associated factor 6		0.09	0.11	0.14		-0.39	-0.42	-0.45	0.13	0.35	0.40	0.44	0.33	0.13	0.16	0.18		-0.40	-0.35	-0.31	-0.38	-0.10	0.00	0.06
FBgn0038948	PSR	phosphatidylserine receptor	-0.87	-0.21	-0.28	-0.29		-1.38	-1.31	-1.31		0.03	-0.04	-0.04		0.02	-0.02	-0.02	3.12	0.11	0.26	0.26	0.31	0.92	0.93	0.90
FBgn0035402	Usp5	Ubiquitin specific protease 5	-0.28	-0.22	-0.19	-0.18	-0.47	0.11	0.00	-0.03		-0.07	0.00	0.03		0.36	0.41	0.41	1.24	0.12	0.17	0.21	0.78	0.94	1.04	1.09
FBgn0003416	sl	small wing		1.30	1.37	1.48		0.13	0.04	-0.03	0.30	0.31	0.37	0.49		-0.24	-0.22	-0.18		-0.61	-0.73	-0.63		0.16	0.11	0.27
FBgn0030018	slpr	slipper		0.16	0.20	0.31	0.32	0.22	0.16	0.09	0.11	0.00	0.06	0.16		-0.23	-0.24	-0.19		-0.32	-0.44	-0.35		0.20	0.18	0.33
FBgn0033773	Mos	Mos oncogene		1.35	1.26	1.21		2.77	2.90	2.90	1.39	1.69	1.70	1.69												
FBgn0029778	RhoGAP5A	Rho GTPase activating protein at 5A	-0.04	-0.13	-0.20	-0.20	-0.51	0.01	0.09	0.09	-0.09	-0.11	-0.10	-0.10	0.31	0.06	0.03	0.03	0.17	-0.16	0.04	0.04	0.24	-0.13	-0.10	-0.10
FBgn0262866	S6kII	Ribosomal protein S6 kinase II		0.32	0.33	0.42		-0.64	-0.67	-0.74		-0.05	0.03	0.13		-0.03	-0.01	0.03		0.38	0.26	0.34		0.29	0.27	0.41
FBgn0015402	ksr	kinase suppressor of ras		0.05	0.10	0.18		0.27	0.23	0.17		0.15	0.22	0.30		-0.26	-0.24	-0.20		0.11	0.00	0.08		0.04	0.03	0.15
FBgn0041100	park	parkin		-0.05	-0.18	-0.18										0.00	-0.02	-0.02		0.83	1.03	1.03		-0.08	-0.11	-0.13
FBgn0030748	Traf-like	TNF-receptor-associated factor-like		-0.59	-0.49	-0.49										-0.16	-0.15	-0.15		-0.40	-0.23	-0.23				
FBgn0004360	Wnt2	Wnt oncogene analog 2																						0.53	0.65	0.65
FBgn0037431	CG17917																			0.63	0.48	0.40				
FBgn0038973	Pebp1	Phosphatidylethanolamine-binding protein 1										-0.20	-0.44	-0.51												
